# Open anterior hip dislocation combined with infection and injury of the common femoral artery and vein: A case report and literature review

**DOI:** 10.1097/MD.0000000000042623

**Published:** 2025-06-20

**Authors:** Jia-Bao Jiang, Zhou Zhong, Yi-Jie Yin, Sujan Shakya, Hao Liu, Yi Li, Zhou Xiang, Jia-Lei Chen

**Affiliations:** a Department of Orthopedics Surgery, Orthopedic Research Institute, West China Hospital, Sichuan University, Chengdu, China; b Department of Orthopedics Surgery, West China Sanya Hospital, Sichuan University, Sanya, Hainan, China.

**Keywords:** anterior hip dislocation, case report, deep vein thrombosis, open fracture, vascular injury

## Abstract

**Rationale:**

Open anterior hip dislocation resulting in vascular injury is extremely rare and is a severe orthopedic emergency that can potentially lead to limb necrosis and even life-threatening consequences.

**Patient concerns:**

A 51-year-old woman presented to our hospital with an open anterior dislocation of the left hip secondary to a fall from height. The patient had a severe infection in the left inguinal area; at presentation, there was dislocation of the femoral head lasting for the past 5 days. Doppler ultrasound revealed left common femoral vein thrombosis.

**Diagnoses:**

The patient was diagnosed with a multiple-injury patient with an acetabular fracture, femoral head fracture, and open anterior dislocation of the hip with vascular injury and infection.

**Interventions:**

After placement of an inferior vena cava filter, the reduction was performed under general anesthesia. The common femoral artery ruptured during the process. After suturing, continuous drainage was maintained. During the subsequent treatment, the infection gradually eroded the common femoral artery and formed a pseudoaneurysm. Lower limb vascularity was restored by autologous saphenous vein grafting, repeated debridement, and sensitive antibiotics.

**Outcomes:**

The infection of the patient was gradually controlled, and the lower limb was successfully preserved. During the 1-year follow-up, the patient returned to normal life without disability.

**Lessons:**

From this rare case, we suggest that a therapeutic approach should be evaluated after adequate exclusion of deep vein thrombosis to prevent thromboembolic events. Moreover, gentle reduction operation, thorough debridement, and timely infection control are essential to prevent complications and improve the functional prognosis of patients.

## 1. Introduction

Traumatic hip dislocations are extremely rare and are usually caused by high-energy trauma, commonly seen in traffic accidents and high fall injuries. Anterior hip dislocations are less common, accounting for ≈10% of all dislocation types.^[1]^ In addition, open anterior hip dislocations are much rarer. The transmission of strong violence leads to lacerations of soft tissue and skin, usually accompanied by skeletal injuries including fractures of the femoral head, neck or shaft, acetabulum, and/or pelvis.^[2]^ According to the position of the femoral head, it can be classified as superior dislocation, in which the femoral head is displaced to the iliac or pubic region, and inferior dislocation, in which the femoral head is located in the obturator region.^[3]^ In superior anterior dislocation, the femoral head is located at the level of the pubic bone, which may result in injury to the neurovascular bundle and tearing of the pubococcygeus and iliopsoas muscles. Common complications include avascular necrosis of the femoral head and suppurative arthritis, which are significant threats to the patient’s prognosis; vascular injury due to open anterior hip dislocation is extremely rare, which is a serious orthopedic emergency that may lead to necrosis of the patient’s limb or even life-threatening. However, the treatment of this aspect is not currently well elaborated in the literature, with only a few cases reported. Herein, we describe a rare case of a patient with open anterior hip dislocation combined with injury to the common femoral artery and vein. Following several surgical procedures, the patient was discharged and successfully returned to normal life. Due to the rarity of this type of injury, there is no standard treatment protocol. The purpose of this study was to analyze the mechanism of injury, discuss the treatment protocol, provide a comprehensive review of hip dislocation combined with vascular injury in the current literature, and finally provide better treatment options.

## 2. Case report

**Figure 1.**
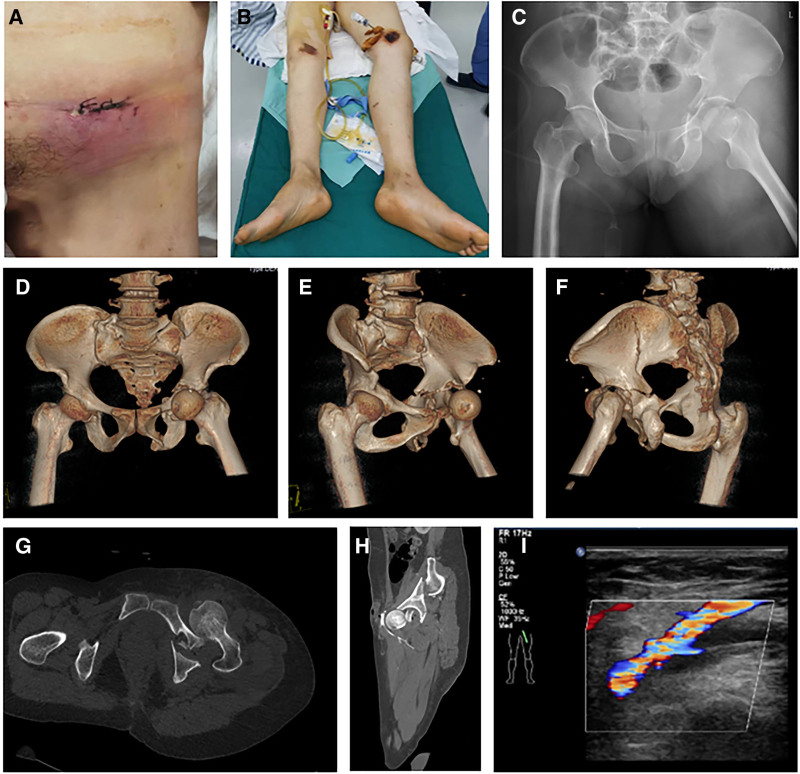
(A) The appearance of the patient’s left inguinal area wound on admission. (B) The patient’s left lower limb was in external rotation deformity before reduction. (C–F) The X-ray and computed tomography (CT) images on admission showed the femoral head in anterior dislocation and acetabular fracture (letournel-judet T shaped). (G) The CT tomography image on admission showed an avulsion fracture of the femoral head. (H) The CT angiography image on admission showed the dislocated femoral head compressing the common femoral artery. (I) The Doppler ultrasound image of the lower limb veins on admission showed a filling defect of the blood flow signal in the common femoral vein, indicating thrombosis.

A 51-year-old female patient presented to our hospital complaining of pain, bleeding, and limited mobility in the left hip joint after falling from a height for 5 days. The patient underwent emergency debridement and suturing at the external hospital. Intraoperative exploration revealed a wound of ≈5 cm in the left inguinal region, within which the femoral head was visible under direct vision. In the subsequent days, the patient’s wound exhibited a gradual onset of erythema, edema, and purulent discharge. The patient presented with hyperthermia upon referral to our emergency department, exhibiting elevated skin temperature surrounding the wound site, significant erythema, and exudate (Fig. 1A). The patient underwent left femoral supracondylar skeletal traction; on physical examination, the patient’s left lower limb was in external rotation deformity (Fig. 1B). Pelvic separation and compression test yielded positive sign, while the neurovascular examination at the distal end of both lower limbs was normal. Radiographic (X-ray and computed tomography [CT]) evaluation on admission revealed an anterior dislocation of the left hip (superior type), acetabular fracture (letournel-judet T shaped), femoral head fracture (Pipkin IV; Fig. 1C–G), and compression of common femoral artery (Fig. 1H); Doppler ultrasound of the arteries and veins of the lower limbs identified a thrombosis of the left common femoral vein (Fig. 1I).

**Figure 2.**
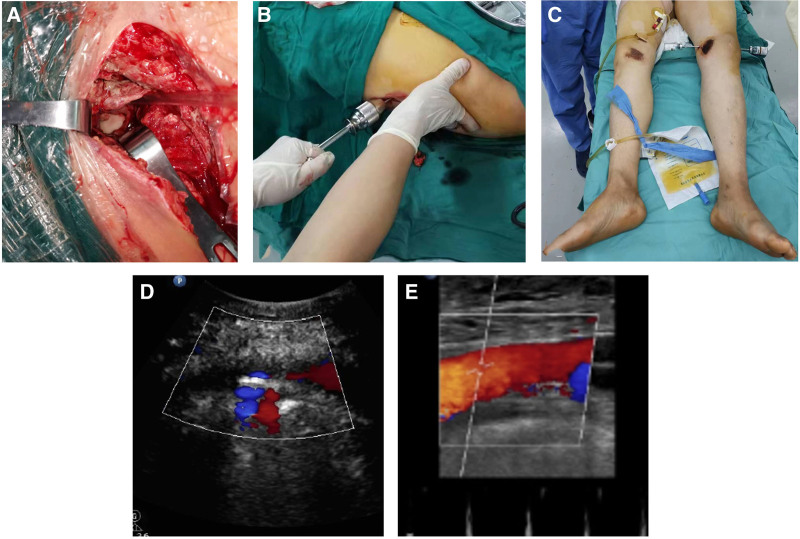
(A) Exploration of the wound revealed a dislocated femoral head with a severe infection of the femoral vascular sheath. (B) Schanz nail was used to assist reduction intraoperatively. (C) The patient’s left lower limb external rotation deformity was corrected after reduction. (D) The intraoperative Doppler ultrasound image showed a lumen-filling defect in the left common femoral artery at the suture. (E) Recovery of luminal blood flow after incision and removal of the thrombus.

The patient underwent emergency surgery under general anesthesia. First, the vascular surgeon placed an inferior vena cava filter (IVCF). Then, we performed debridement of the wound in the left inguinal region; after removal of the original sutures, a large amount of purulent exudate was evacuated from the wound, revealing a large amount of pus, visible on the surface of the tissue. Deep secretions were taken for culture. After repeated irrigation, a rupture of the joint capsule in front of the hip joint was observed, along with dislocation of the femoral head (Fig. 2A). There was concomitant severe contusion of the cartilage of the femoral head, as well as a small amount of fragmented bone. After removing the fragments, we failed in several attempts to reset the femoral head by manipulation. The Schanz nail was inserted into the femoral neck along the lateral aspect of the femur (greater trochanter), and the proximal end of the femur was pulled against it (Fig. 2B). A clear sense of resetting was felt, and intraoperative fluoroscopy confirmed that the hip reduction was successfully achieved; the patient’s external rotation deformity of the left lower limb was corrected (Fig. 2C). However, it was observed that there was significant blood oozing from the femoral vascular sheath, along with a rupture of the common femoral artery measuring ≈2 mm in length. Following systemic heparinization, the vascular surgeon performed suturing to repair the ruptured vessel. However, after the suturing was completed, the patient’s left dorsalis pedis artery pulse was absent, resulting in a cold lower limb. Intraoperative Doppler ultrasound revealed slow blood flow in the left lower limb and a lumen-filling defect in the left common femoral artery at the suture site (Fig. 2D). Re-exploration revealed thrombosis proximal to the rupture in the common femoral artery. After removing the thrombus and suturing the vessel, the left dorsalis pedis artery resumed pulsation, and ultrasound revealed restoration of intravascular blood flow (Fig. 2E). A deep drainage tube was placed, and rubber drainage strips were placed subcutaneously.

In the postoperative setting, we empirically used intravenous piperacillin sodium tazobactam sodium (4.5g q8h) for infection control and enoxaparin sodium (0.4ml q12h subcutaneously) for anticoagulant treatment. The patient’s systemic hyperthermia symptoms (fever) gradually improved, but there was still purulent secretion oozing from the left inguinal region. The patient’s C-reactive protein, interleukin-6, and erythrocyte sedimentation rate remained above the normal values. The results of secretion culture reported the presence of *Escherichia coli* and *Serratia marcescens* mucoid subspecies; the drug sensitivity test was sensitive to piperacillin sodium tazobactam sodium. Therefore, we continued treatment with current antibiotics. One week later, we performed a second wound debridement; an intraoperative exploration revealed an encapsulated pus accumulation in the deep soft tissue. There was severe adhesion around the femoral vascular sheath, while the pulsation of the common femoral artery was normal; there was no rupture of the common femoral artery or vein.

**Figure 3.**
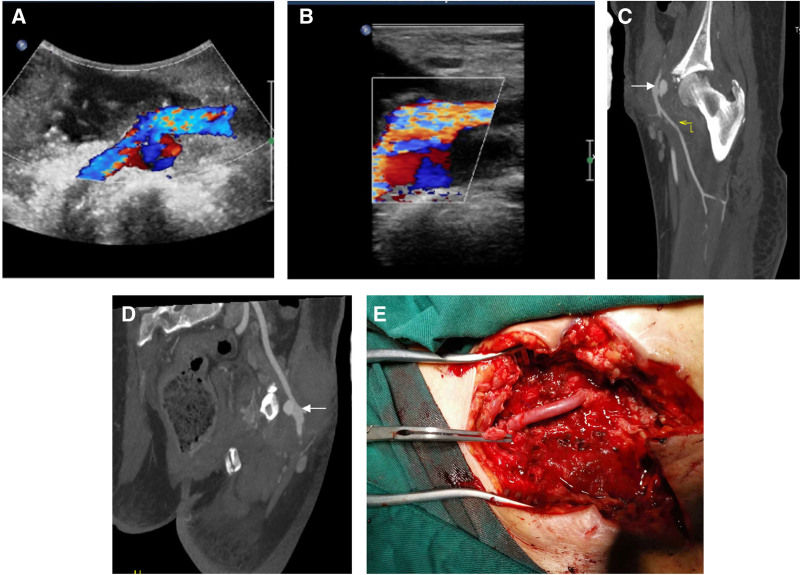
(A and B) The Doppler ultrasound image revealed a pseudoaneurysm formation in the left inguinal region with lumen narrowing and local intravascular thrombosis. (C and D) The computed tomography arteriography of the lower limbs revealed contrast leakage forming enhanced nodular shadows (white arrows), and active bleeding due to vascular rupture was considered. (E) After autologous saphenous vein grafting, there was no significant bleeding from the anastomosis and the artery pulsed well.

The patient’s drainage of the wound gradually decreased with the prolongation of the treatment time. However, erythema and oozing at the wound still existed, and there was no obvious decreasing trend of inflammatory markers. Two weeks later, a second weakness of the dorsalis pedis artery pulse was observed with the cold left lower extremity. Emergency vascular ultrasound revealed a pseudoaneurysm formation in the left inguinal region with lumen narrowing and local intravascular thrombosis (Fig. 3A and B). A CT arteriography (CTA) of the lower limb demonstrated contrast leakage; therefore, a diagnosis of vascular rupture was considered (Fig. 3C and D). Emergency surgical treatment was performed. Intraoperative exploration revealed severe infection and adhesions around the common femoral artery within the wound. An 8-mm rupture of the common femoral artery was visible, involving the superficial and deep femoral arteries. The vessels within 2 cm of the rupture were eroded by abscesses, and the walls of the vessels were inelastic and difficult to suture. After systemic heparinization, the vascular surgeon resected the eroded artery, resected the right saphenous vein for about 5 cm, and performed bypass grafting of the common femoral artery, superficial artery, and deep femoral artery. At the end of the anastomosis, the vessels pulsed regularly, with no significant blood leakage from the wound (Fig. 3E); wound drainage was placed, and the wound was sutured.

**Figure 4.**
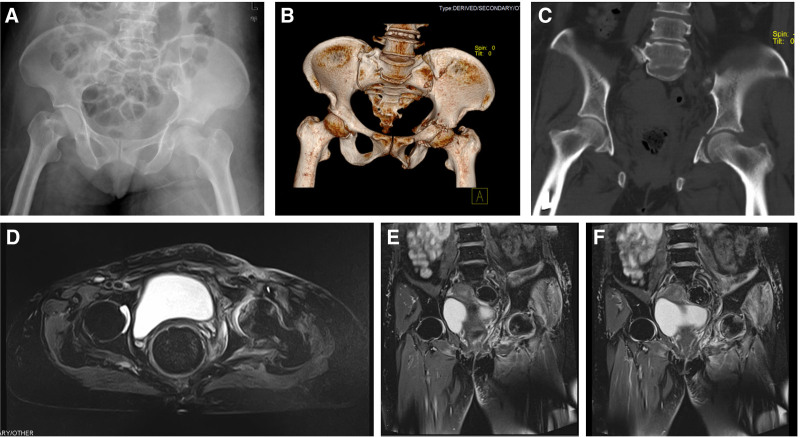
(A–C) The X-ray and computed tomography images showed that the left femoral head was in place, and the fracture site was not significantly displaced, with local bone callus growth. (D–F) The magnetic resonance imaging showed abnormal signals in the left femoral head, and the high signals were mainly located at the edge of the femoral head, considering the contusion.

Within 1 week following the surgery, the patient’s deep wound drainage flow gradually decreased; the redness and swelling of the wound gradually subsided. After removing the drainage tube, a repeated examination of the lower limb vascular ultrasound showed that the lumen of the left common femoral artery was fully irrigated, and no obvious thrombus was seen in the lower limb veins. The vascular surgeon evaluated and decided to remove the IVCF. Repeat X-ray and CT showed that the left femoral head was in position, and the fracture site showed bone callus formation (Fig. 4A–C). After repeated fresh dressing for the wound, the exudate gradually decreased, and the wound healing was initiated. During this period, cultures of wound secretions were frequently taken, and no bacterial growth was cultured.

Six weeks after the patient was admitted to our hospital, magnetic resonance imaging of the hip was performed after removing the patient’s left femoral condylar supracondylar traction. The results revealed abnormal signals in the left femoral head, mainly in the femoral head rim, and contusion was considered (Fig. 4D–F).

**Figure 5.**
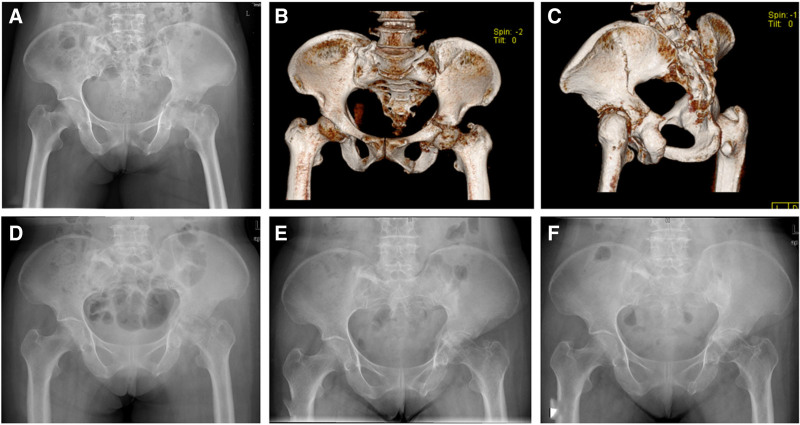
(A–C) The X-ray and computed tomography images on discharge from the hospital. (D–F) The X-ray images at 4 months, 5 months, and 1 year after the injury showed that the fracture was healed with no significant collapse of the femoral head. The left hip joint space was narrowed, and the traumatic hip arthritis was considered.

The patient was processed and discharged from the hospital 2 months after admission; the left inguinal wound completely healed; X-ray and CT reevaluation demonstrated that the femoral head did not present any significant collapse; and the fracture sites were healing with bone callus formation (Fig. 5A–C).

During the postdischarge follow-up, the patient gradually started to walk on crutches after 4 months and unaided 5 months after the injury. One year following the injury, the patient walked and walked up and down the stairs without any obvious pain and resumed normal daily life; with a good Harris score (87). X-ray showed that the fracture line was blurred, and the femoral head did not see any obvious collapse (Fig. 5D–F).

## 3. Discussion

Vascular injuries caused by open anterior hip dislocation are extremely rare, leading to a series of serious complications if the diagnosis is missed. We searched the literature on anterior hip dislocation with vascular injury using electronic databases, including Cochrane, PubMed, Web of Science, and EMBASE. There are 9 cases of this type of injury reported so far, with a high mortality rate and functional disability.^[4–11]^ The characteristics of the relevant literature are summarized in Table 1. Therefore, it is important to understand the pathogenesis of this type of injury to make a clear diagnosis and appropriate treatment plan for orthopedic surgeons.

**Table 1:** Characterization of other cases of anterior hip dislocation with vascular injury in the literature.

Case	Age (yr)/sex	Mechanism of injury	Vascular injury	Associated injury	Time of reduction (h)	Treatment	Follow-up (mo)	Result
Schwartz and Haller^[11]^	5/M	Hit by an automobile	Transection of FA and FV	Laceration from anterior iliac spine to perineum >10 cm	1	Reduction and repair of vessels	12	Limb survived with no evidence of AVN
Bonnemaison and Henderson^[5]^	11/M	Run over by a car	Acute occlusion of the common FA	Laceration of the hip and left femur supracondylar fracture	6	Reduction and strip of the vascular tunica, repeated washes of procaine, and warm normal saline	None	Died 36 h after admission due to abdominal hemorrhage and acute renal failure
Hampson^[7]^	49/M	Traffic accident	Left femoral venous obstruction	Left inferior pubic ramus fracture	3	Reduction	24	No symptoms of the hip joint and radiographs showed no abnormality
Hampson^[7]^	17/M	Collided with a lorry	Right FA was completely divided	Open fracture of the right femur	7	Reduction and right leg was amputated	None	Died 30 h after admission due to PE
Nerubay^[10]^	15/M	Run over by a car	Compression of the common FA and tear of the FV	Bilateral fractures of ischium bone	Unclear	Reduction and repair of vessels	24	Periarticular ossification and AVN of the femoral head
Holt and McCarty^[8]^	40/F	Struck by a car	Thrombosis of the distal right external iliac artery, proximal common FA, superficial FA, and profunda FA; avulsion of the greater saphenous vein, and external iliac vein tear	None	5.5	Reduction and right guillotine below-knee amputation	None	Died 49 d after admission due to sepsis secondary to pneumonia
Amendola et al^[4]^	28/M	Motorbike accident.	PE and compression of right femoral vessels and thrombosis of right posterior tibial veins	Ipsilateral fracture of the greater trochanter of the femur and fracture of the left acetabulum and femoral shaft.	4.5	Anticoagulation therapy, reduction, and intramedullary nail of the left femur	6	Radiographic healing occurred and the patient had a complete recovery
Dieterich et al^[6]^	90/M	Falling at home	Thrombosis of the right external iliac vein extending into the common FV	None	Unclear	Placement of inferior vena cava filter and reduction and anticoagulation therapy	12	No pain and walking without limitation
Jain et al^[9]^	28/M	Motor vehicle accident	Vascular occlusion at popliteus level	Ipsilateral knee dislocation and segmental radius fracture of the left upper limb	Unclear	Reduction and exposed FA and removed the clot	Unclear	Above-knee amputation

AVN = avascular necrosis, FA = femoral artery, FV = femoral vein, PE = pulmonary embolism.

The mechanism of anterior hip dislocation was described in 1950.^[12]^ The mechanism is generally related to 2 types of traumatic forces. The first type is a traffic accident, where the patient’s hip is in an abducted position, the knee is flexed, and the impact is against the front seatback during sudden braking, causing the femoral head to break through and dislocate from the weaker lower anterior part of the hip joint capsule. The second is fall from height, where the femur is extremely abducted and externally rotated; it experiences direct violence, leading to dislocation of the femoral head. In the case of the current study, the mechanism of injury belongs to the second type, where the enormous force resulted in the tearing of the hip joint capsule, medial ligaments, acetabular fracture, avulsion fracture of the femoral head, and laceration of the skin in the inguinal region. The dislocated femoral head compressed the femoral vascular sheath, causing blunt injuries. After the injury, the patient fulfilled the basic elements of the Virchow triad for venous thromboembolism (hypercoagulable state, endothelial damage, and stasis), leading to the formation of left femoral vein thrombosis.^[13]^

Timely reduction, thorough debridement, and broad-spectrum antibiotic administration are recommended for open anterior hip dislocations.^[14]^ It has been reported that the incidence of avascular necrosis of the femoral head is 4.8% if reduction occurs within 6 hours compared with 58.8% if reduction after 6 hours.^[15]^ In our case, the patient was not treated effectively after the injury, and the hip was in a dislocated position for 5 days. Irreversible ischemia may occur in the femoral head, prolonged venous compression and stasis leading to deep vein thrombosis, and severe soft tissue injuries and infections, representing a significant threat to the patient’s survival and prognosis. We did not rush to reduce the hip after the patient was admitted to our hospital, considering that the patient was at a very high risk of thromboembolism formation. After completing CTA and Doppler ultrasound, a left common femoral vein thrombus was detected. The vascular surgeon evaluated and placed an IVCF. Dieterich et al.^[6]^ recommend that operations such as hip reduction should be performed after the risk of deep vein thrombosis has been ruled out. Performing reduction or hip manipulation without ruling out the risk of thrombosis in the lower extremity veins may lead to serious complications such as pulmonary embolism from dislodged thrombus.

We found a rupture of the common femoral artery during exploration after the patient’s hip reduction. A new thrombus was formed immediately after the repair was completed. Although prompt thrombectomy was performed to restore blood supply to the patient’s lower limbs, the patient’s common femoral artery formed a pseudoaneurysm and ruptured during subsequent treatment. Finally, blood supply was restored through autologous vascular grafts, avoiding necrosis and amputation of the limb. We believe that infection in the depths of the wound eroded the vessel, leading to a more brittle vessel. The force of the hip reduction caused the common femoral artery to rupture. Therefore, for patients with anterior dislocation of the hip joint, we recommend that the reduction should be as gentle as possible to avoid secondary damage to the femoral vascular sheath.

Despite suture repair, the infection in the depths of the patient’s wound was not controlled for a shorter duration of time, leading to further vascular damage. There is a high risk of failure due to infection for artificial vessel implantation or autologous bypass grafting at this time.^[16]^ Therefore, timely control of the infection is significant for the survival of the patient’s limb. An intravenous broad-spectrum antibiotic provides significant improvement in systemic symptoms, but local infection control is limited. Although intraoperative debridement can be effective in removing deep necrotic and infected tissues, repeated operations may cause medical damage to local blood vessels. Vacuum-assisted closure therapy is a useful option for severely infected wounds but is contraindicated for wounds after vessel anastomosis.^[17]^ We suggest that local skin/muscle flap graft repair or antibiotic bone cement implantation can be attempted for such wounds for effective infection control.

Matta^[18]^ suggested that acetabular fractures with >3-mm displacement require open reduction and internal fixation to achieve better joint function. However, due to the prolonged period of treatment for the infection in the groin, this patient’s acetabular fracture missed the optimal time for surgery. Moreover, the risk of infection is higher if internal fixation is performed. During the hospitalization, repeated X-rays and CT reevaluation showed no significant displacement of the fracture, and the local bone callus formation was observed. Therefore, we did not conduct internal fixation for this old acetabular fracture.

The magnetic resonance imaging examination was performed 2 months after the patient’s injury and revealed a patchy high signal in the left femoral head, mainly around the femoral head, with no obvious abnormality in the joint space. We believed that this change is a subchondral injury acquired during the dislocation of the joint, just because the center of the femoral head did not show any signal change. During the 1-year follow-up, the patient had a satisfactory hip range of motion and no significant pain, and no significant femoral head collapse was found on the X-ray. Therefore, we reported this case of open anterior dislocation of the hip combined with injury to the common femoral artery and common femoral vein. The patient management was successful, the limb and the entire life were fully preserved, and the patient returned to normal life without significant disability. This article can serve as a reference for guidance on how to effectively manage this rare injury.

Open anterior hip dislocation combined with infection and vascular injury is extremely rare. Attention should be paid to the mechanism of injury and the associated anatomical structures when treating these patients. From this rare case, we recommend completing venous Doppler ultrasound and CTA of the lower limbs for vascular involvement to rule out the risk of thrombosis before attempting hip joint reduction. If necessary, placement of IVCF and anticoagulation are recommended. Furthermore, gentle reduction operation is important to avoid secondary damage. In addition, thorough debridement and timely infection control are essential to prevent complications and improve functional outcomes in patients.

## Author contributions

**Conceptualization:** Jia-Bao Jiang, Zhou Xiang, Jia-Lei Chen.

**Data curation:** Jia-Bao Jiang, Zhou Zhong, Yi-Jie Yin, Sujan Shakya, Hao Liu, Yi Li.

**Methodology:** Jia-Bao Jiang, Zhou Xiang.

**Writing – original draft:** Jia-Bao Jiang.

**Writing – review & editing:** Zhou Xiang, Jia-Lei Chen.

**Supervision:** Jia-Lei Chen.
